# Polycyclic Aromatic Hydrocarbons (PAHs) in Roasted Pork Meat and the Effect of Dried Fruits on PAH Content

**DOI:** 10.3390/ijerph20064922

**Published:** 2023-03-10

**Authors:** Sylwia Bulanda, Beata Janoszka

**Affiliations:** Department of Chemistry, Faculty of Medical Sciences in Zabrze, Medical University of Silesia in Katowice, Jordana 19, 41-808 Zabrze, Poland

**Keywords:** PAHs, HPLC, meat roasting, dried fruits, cancer prevention, diet

## Abstract

Diet is one of the main factors affecting human health. The frequent consumption of heat-treated meat has been classified as both directly carcinogenic to humans and as a risk factor, especially in the case of cancers of the gastrointestinal tract. Thermally processed meat may contain harmful muta- and carcinogenic compounds, including polycyclic aromatic hydrocarbons (PAHs). However, there are natural ways to reduce the risk of diet-related cancers by reducing the formation of PAHs in meat. The purpose of this study was to determine changes in PAH levels in pork loin dishes prepared by stuffing the meat with dried fruits (prunes, apricots and cranberries) and baking it in a roasting bag. High-performance liquid chromatography with fluorescence detection (HPLC-FLD) was used to conduct a quantitative analysis of seven PAHs. Recovery results ranged from 61 to 96%. The limit of detection (LOD) was 0.003 to 0.006 ng/g, and the limit of quantification (LOQ) was 0.01 to 0.02 ng/g. Gas chromatography–mass spectrometry (GC-MS/MS) was used to confirm the presence of PAHs in food. The total PAH content of the roasted pork loin was 7.4 ng/g. This concentration decreased by 35%, 48% and 58% when the meat was roasted with apricots, prunes and cranberries, respectively. The cranberries also inhibited the formation of benzo(a)pyrene to the greatest extent. Thermally treating meat stuffed with dry fruits may be a simple and effective way to prepare foods with reduced levels of mutagens and carcinogens belonging to PAHs, and thus reduce the risk of cancer.

## 1. Introduction

With rapid developments in both population and lifestyle, cancer has become the second leading global non-infectious disease [1,2]. After cardiovascular disease, cancer is the second leading cause of death in the world [3,4].

The International Agency for Research on Cancer (IARC), an intergovernmental agency of the World Health Organization (WHO), published a report in 2020 in which 19.30 million new cancer cases and 10.00 million deaths were reported. Breast cancer is the most frequently diagnosed cancer, followed by lung cancer, colorectal cancer and prostate cancer, with incidences of 11.7%, 11.4%, 10.0% and 7.3%, respectively. The WHO predicted that, by 2040, the increase in the number of diagnosed cancers may have increased to 28.40 million. Therefore, there is an urgent need to develop approaches or strategies based on cancer prevention mechanisms [2,5]. The aforementioned colorectal cancer and other cancers, such as prostate, breast, stomach and pancreas cancers, are associated with the excessive consumption of thermally processed red meat [2,6]. This is a reason that the IARC classified processed meat as “carcinogenic to humans” (Group 1) and red meat as “probably carcinogenic to humans” (Group 2A) in 2015 [7].

During thermal processing of meat, some genotoxic, mutagenic and carcinogenic compounds may be formed, such as polycyclic aromatic hydrocarbons (PAHs) [8,9]. PAHs are a group of 200 chemical compounds comprising several condensed aromatic rings [10,11]. Compounds with more than four rings are more stable and toxic [9,12].

Continuous environmental and dietary exposure to PAHs implies genotoxic and carcinogenic potential for humans. Mutations in DNA replication caused by the metabolic activation of PAHs in mammalian cells to diol epoxides appear to be the initial process of the development of cancer. Non-genotoxic effects of PAH, such as increased blood pressure, lipid/lipoprotein abnormalities and insulin resistance, have also been reported [12,13].

PAHs in food can come from a polluted environment or be formed during thermal processing, such as in meat during smoking and grilling [9,14]. Due to their harmful effects, PAHs are determined in a variety of food products [10,15]. Studies confirm that PAHs are present not only in thermally processed meat, but also in smoked cheese [16], coffee and tea [17], edible oils [18,19], toasted bread [20] and even yogurt and milk [21].

There have been remarkable changes in the treatment of cancer patients over the last several decades, and the survival rate of these patients has increased significantly. These changes can largely be attributed to the improvement of surgical treatment, the development of chemotherapy and radiotherapy, and the emergence of modern targeted therapies [22,23]. 

Cancer prevention is no less important than the process of cancer treatment itself, which is why ways to reduce human exposure to carcinogenic compounds continue to be researched. In recent years, knowledge of natural products with chemopreventive effects has been developing. Numerous natural products in the diet (such as phenols, flavonoids, alkaloids, carotenoids and organosulphur compounds) are able to regulate physiological functions and stop carcinogenesis at its various stages [5,24].

One of the new possible methods of diminishing the amount of PAHs in foods was indicated by the results of research by Shariatifar et al., which demonstrated that lactic acid bacteria (live and inactivated strains) are able to remove selected PAHs from aqueous media [25]. The results of this study can also be used to evaluate the effect of oral administration of probiotic supplements on the reduction in PAHs in humans and may serve as a new strategy in the detoxification of PAHs from the diet.

In the countries of the European Union (EU), there are regulations specifying the permissible concentrations of PAHs in some foodstuffs. These regulations are constantly updated in accordance with the results of numerous studies on the determination of PAHs in food. In 2006, one of the hydrocarbons—benzo(a)pyrene (BaP)—was indicated by the EU Scientific Food Committee (SFC) as a marker of the occurrence of PAHs and the carcinogenic effect of this group of compounds in food [26]. However, after laboratory testing of many different food samples, the European Food Safety Authority (EFSA) found other carcinogenic and genotoxic PAHs in many samples, despite BaP being negative [27]. Based on these data, the EFSA Scientific Panel on Contaminants in the Food Chain concluded that benzo(a)pyrene is not a suitable marker for polycyclic aromatic hydrocarbons in food and that the system of four compounds (PAH4): BaP, benzo(a)anthracene (BaA), benzo(b)fluoranthene (BbF) and chrysene (Chr), or eight compounds (PAH8): PAH4 + benzo(k)fluoranthene (BkF), benzo(ghi)perylene (BghiP), dibenzo(ah) anthracene (DiBahA) and indeno(1,2,3-cd)pyrene (IP), would be the most appropriate indicators of PAH concentration in food [28]. On the other hand, when classifying PAHs, the carcinogenic properties of each compound are taken into account. BaP is classified by IARC as an agent from Group 1, and the remaining three PAH4, as well as BkF and IP, are classified as agents from Group 2B (i.e., possibly carcinogenic to humans). DiBahA belongs to Group 2A.

According to the regulations, the concentrations of BaP and PAH4 in smoked meat products should not exceed 5 μg/kg and 30 μg/kg, respectively, and from 1 September 2014 it should be reduced to 2 μg and 12 μg/kg [28]. In addition, the regulations state that heat-treated (grilled, barbecued) meat and meat products sold to the final consumer must not contain more than 5 µg of BaP and 30 µg of PAH4 in one kg. 

PAHs can be formed in meat from the natural existing organic molecules in high-protein foods: aliphatic α-amino acids; reducing sugars, mainly D-glucose; fatty acids and fats; and cholesterol and plant sterols [14,29]. These food components are easily fragmented at high temperatures (e.g., above 200 °C) during pyrolysis, and the resulting free radicals can form PAHs through pyrosynthesis [29,30]. 

Although the concentrations of PAHs in food products are in the order of μg/kg, i.e., they are not high, frequent, systematic consumption of processed meat products means that exposure to PAHs may become harmful to human health. Therefore, many studies have aimed to identify simple, natural ways to prepare meat dishes with a limited content of carcinogenic compounds from the PAH group [30,31,32,33,34]. Of particular note is the use of antioxidant-rich supplements as ingredients added to heat-treated meats. These are compounds present in natural products, such as spices or vegetables [35,36,37,38], and also in different marinades prepared with beer, wine or tea [32,39,40,41,42]. The results of some studies indicate a positive relationship between the reduction in PAH concentration and the antioxidant potential of the additives used [37,38,39,40]. 

The detailed effect of various natural additives on the formation of PAHs in heat-treated meats of various types (chicken, beef and pork) has been discussed and compiled in the form of a table in our review paper [43]. Among the additives that particularly intensively affect the inhibition of PAHs, it is worth highlighting marinades containing phenolic compounds from green tea (reduction of BaP and total PAHs by 71 and 55%, respectively) [42], beer marinades (78%—BaP and 67%—PAH8 for Heineken beer) [40] and vinegars (87%—BaP under the influence of white wine vinegar; 82%—PAH4, elderberry vinegar) [32], as well as natural spices, such as black pepper, garlic, ginger, onion, paprika and red chilli [44]. Under the influence of ginger, BaP can be reduced by 100% in fried beef [44].

However, there are studies that indicate the opposite effect of plant additives on the formation of PAHs. For example, no effect of the addition of curcuma and cinnamon powder on the content of PAHs in pork jerky was found [45], and an increase in BaP and PAH8 under the influence of two types of alcoholic beer was noted [40].

A review of the literature [43] shows that few studies have so far considered the effect of the components contained in fruit on the formation of PAHs [32,40]. These involve different fruit vinegars [32] and apple polyphenol extract [46].

In Poland, various fruits, both raw and dried, are often added to cooked meats. They are usually used as a filling for dishes and eaten together with meat. To the best of our knowledge, the effect of fruit addition on the formation of PAHs has not been studied so far. Therefore, the aim of our research was to determine the content of PAHs in meat subjected to electric oven roasting using roasting bags and to study the effect of dried fruit (cranberries, apricots and prunes) on the formation of PAHs in dishes made from pork loin. Seven compounds belonging to PAH8, including all of the PAH4 group, were selected for the study. The obtained results may be an indication of how to minimize human exposure to carcinogenic compounds from the diet through simple methods of preparing meat dishes.

## 2. Materials and Methods

### 2.1. Chemicals and Materials

PAH standards: The following polycyclic aromatic hydrocarbons were used in the study: benzo(a)anthracene (BaA), chrysene (Chr), benzo(a)pyrene (BaP), benzo(b)fluoranthene (BbF), benzo(k)fluoranthene (BkF), dibenzo(ah)anthracene (DiBahA) and benzo(ghi)perylene (BghiP). The structures of these compounds are shown in Table 1.

Standard substances (purity > 98%) were bought from Sigma Aldrich (Steinheim, Germany). Standard stock solutions of PAHs (each 0.2 mg/mL) were used to prepare standard mixtures of 1 µg/mL concentration in acetonitrile. Solutions at different concentrations of 0.05, 0.1, 0.15, 0.2, 0.5, 1, 2, 5, 20, 50, 100 and 200 ng/mL were prepared by diluting the standard mixture. These solutions were used to establish detection limits and construct standard calibration curves. 

Other chemicals: The HPLC-grade organic solvents used for extraction and for the mobile phase preparation, toluene, dichloromethane, n-hexane and acetonitrile, were purchased from Avantor™ Performance Materials (Gliwice, Poland). Water was obtained from a simplified water purification system (Millipore, Vienna, Austria). Sodium hydroxide (analytical-reagent grade) for alkaline hydrolysis of the samples was bought from Avantor™ Performance Materials (Gliwice, Poland). Diatomaceous earth extraction columns (Extrelut, 20 mL) and refill materials were obtained from Merck (Darmstadt, Germany). Solid-phase extraction (SPE) columns filled with propyl sulfonic acid (SPE-PRS, 500 mg, 3 mL) were bought from J.T. Baker (Avantor™ Performance Materials BV, Deventer, The Netherlands). These columns were preconditioned with dichloromethane (4 mL). Silica gel (70–230 mesh) used for column chromatography was obtained from Merck (Darmstadt, Germany). It was activated at 200 °C for 12 h.

### 2.2. Preparation of Meat Dishes—Pork Loin Stuffed with Dried Fruits

Meat samples from pork loin dishes were investigated. Three types of roasts stuffed with dried fruits and one without additives (control sample) were cooked. A tradition of Polish cuisine is roasting meat with the addition of various fruits, whether dried or fresh. The following dried fruit fillings were used in the study: prunes, apricots and cranberries. 

The dried fruits were bought in quantities of 1 kg (5 packs of 200 g each) in a local market. Apricots and cranberries were of German production. Apricots, as described on the packaging, were preserved with SO_2_ and cranberries with sorbic acid (hexa-2,4-dienes acid). Prunes were of Polish production and did not contain preservatives.

Fruits of each kind were crushed with a knife and a blender. Then, 200 g of each fruit were stuffed into 1 kg of pork loin. The research material (meat) was ordered from a local butcher as two pork tenderloins from one pig. The meat weighed over 4 kg in total. Bones and a few small pieces of fat were excised before the dishes’ preparation. The pork loin was divided into four portions of 1 kg each. In three portions, cuts were made with a knife on both sides, so that a hole with a diameter of 3 cm was created in the depth of the tenderloin. Each of these holes was tightly filled with ground dried fruit of a given type (200 g/kg of pork). The fourth portion was left without fruit. Each dish prepared from pork loin had the following dimensions: perimeter 18 cm, width 10 cm and length 20 cm. Then, each was wrapped in aluminum foil and stored at 4 °C for 12 h. After this time, the aluminum foil was removed, and each piece of meat was placed separately in a roasting bag. Both ends of the pouch were sealed off. The meat did not touch the bag from the inside. 

### 2.3. Thermal Processing of Meat

Each portion of the prepared pork loin in a roasting bag was placed in the center of a baking tray and placed in an electric oven preheated to 200 °C. The pork loin without additives and those with prunes, apricots or cranberries were roasted separately at 200 °C for 30 min and then at 180 °C for 60 min. Ten minutes before the end of roasting, the bag was cut open to brown the roast. The meat was unwrapped from the roasting bag after cooling. The fruit additives were carefully removed from the inside of the roast with a knife. The meat from each dish was ground twice using an electric meat grinder. The following weights of pork roasts were obtained from 1 kg of raw meat: 572 g when cooked with dried cranberries, 570 g when prepared with apricots, 552 g with prunes and 520 g when prepared without additives. 

### 2.4. Extraction of PAHs Fraction from Meat Samples 

The procedure applied to isolate PAHs was used by us previously for other meat samples [47,48] and is presented in the form of a diagram in the Supplementary Materials (Figure S1). It is a modification of a clean-up method used by Rivera et al. for the analysis of grilled meat extracts [49]. The procedure is a part of a multi-step analytical process that allows the simultaneous isolation of PAHs, their nitrogenous heterocyclic derivatives (azaarenes) and heterocyclic aromatic amines from a complex matrix of meat samples [48,50]. The first stage of the experiment was the homogenization and alkaline hydrolysis of meat samples. Therefore, 90 mL of NaOH solution (1 mol/L) was added to 30 g of the meat sample and mixed in a homogenizer (Med. Instruments, Warsaw, Poland) for 3 h. From the dense hydrolysate, six portions were weighed out at 20 g each. There was 5 g of meat in each serving. Two units of the hydrolysate were spiked with a standard mixture containing seven PAHs, resulting in samples enriched with 10 ng/g and 40 ng/g of meat. Then, each of the six portions of the alkaline hydrolysate was subjected to a multi-stage extraction. A total of 10 mL of 1 mol/L NaOH and 15 g of Extrelut were added to each portion of the hydrolysate. The mixture was stirred and then introduced into a 20 mL polypropylene column. A total of 60 mL of CH_2_Cl_2_ containing 5% toluene was used for the elution of organic compounds, including PAHs, from the Extrelut column. This extract flowed directly through the phase of the SPE-PRS column, on which nitrogen heterocyclic food contaminations were adsorbed and thus separated from PAH fractions. Due to the low concentrations of PAHs in meat, four dichloromethane extracts obtained from a given meat hydrolysate (without added standards) were combined to obtain a more concentrated sample. This gave extracts equivalent to 20 g of cooked meat. The solvent was evaporated from the extracts to a volume of about 0.5 mL and the rest was removed with a stream of nitrogen. The residue was dissolved once more in 1 mL of n-hexane and then applied on silica gel (10 g) placed in a glass column. For elution, a volume of 25 mL of n-hexane (discarded) and 60 mL of n-hexane–CH_2_Cl_2_ (60:40; *v*/*v*) mixture were used. The n-hexane–CH_2_Cl_2_ mixture contained the PAHs isolated from the meat sample. After solvent evaporation, the obtained residues were dissolved in 500 µL acetonitrile (unspiked and spiked samples) before the HPLC analysis.

### 2.5. Determination of PAHs by HPLC and Fluorescence Detection (FLD)

Quantitative analysis of PAHs was performed using the HPLC Ultimate 3000 TSL analytical system from Dionex Softron (Germering, Germany). It was equipped with a autosampler (WPS-3000 TSL, Dionex Softron, Germering, Germany), a column compartment (TCC-3200, Dionex Softron, Germering, Germany) and a fluorescence detector (FLD) (Shimadzu RF-2000, Kyoto, Japan). Chromeleon software (version 6.80 SP2 Build 2284, Dionex Softron, Germering, Germany) was used to set analytical parameters and collect data. For the separation of PAHs, a Hypersil Green PAH column (particle size 5 µm; 250 × 4.6 mm I.D), with a guard column (5 µm, 10 × 4 mm) from Thermo Scientific (Waltham, MA, USA) was used. The separations were performed under isocratic conditions by using a mixture of acetonitrile and water (84:16, *v*/*v*) as a mobile phase. The flow rate was 1.0 mL/min. The chromatographic column temperature was set to 40 °C. To obtain signals of maximum intensity on chromatograms for the determined PAHs, fluorescence detection was performed by applying excitation (Ex) and emission (Em) wavelength program optimized for the determined compounds. The following Ex/Em wavelength program was used to determine the PAHs: 267/410 nm from 0 to 12.30 min (determination of BaA and Chr), 225/420 nm from 12.31 to 16.20 min (BbF and BkF), 376/410 nm from 16.21 to 18.40 min (BaP) and 300/420 nm from 18.41 to 30.00 min (DiBahA and BghiP). 

The detection of PAHs in the fractions separated from meat samples was performed by comparing the retention times recorded for standards with the values of appropriate components identified in the spiked and unspiked meat samples run under the same conditions. Quantitative analysis was carried out from the fluorescence data, measuring the peak area and using the external calibration curve method.

### 2.6. Limits of Detection and Quantification, Calibration Plots, Repeatability and Reproducibility, and Recovery of PAHs from Meat Matrix

**Figure 1:**
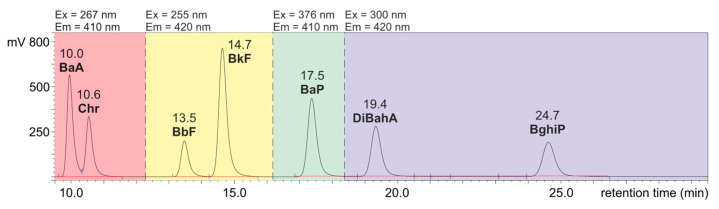
HPLC-FLD chromatogram of PAH standard mixture (injection: 1 ng/10 µL acetonitrile).

Using the HPLC system and a Hypersil Green PAH column, which is specifically designed for the analysis of polycyclic aromatic hydrocarbons, the components of a mixture containing seven PAHs were separated. The results of the separation of these PAH standards are shown as a chromatogram in Figure 1.

Application of the wavelengths λex/λem program enabled us to determine the PAHs at a low concentration level. The selection of wavelength was based on published data [40,47] and the results of PAH standards analysis. 

The limits of detection (LOD) based on the signal-to-noise ratio (S/N = 3) were from 0.1 ng (for BaA and BkF) to 0.2 ng/mL (BbF) for 10 µL injection onto the column. Three times the LOD was defined as the limit of quantification (LOQ) [51]. Quantitative analysis of PAHs was performed on the basis of linear calibration plots recorded in the range of 0.5 to 200 ng/mL, when 10 µL of standard mixture (in acetonitrile) was injected into the column for all PAHs, except for BbF, for which the plot was formed in the range from 1.0 to 200 ng (Table 1). Calibration graphs were constructed using the least-squares method for eight points of concentration. The regression coefficients r for the curves were above 0.999. The lowest concentrations of PAHs that could be determined in meat samples were 0.01 ng/g of cooked meat for BaA and BkF (Table 2).

For quality assurance and control, each batch of meat samples determined by HPLC-FLD was preceded by three consecutive analyses: of acetonitrile, PAH standards solution of 1 ng/10 µL concentration and repeat acetonitrile. Determinations of samples of a given type, i.e., PAH fractions isolated from meat without fruit or with a certain type of additive, were performed within a single batch, whereby the analysis of the real samples was followed by measurements for the corresponding spiked samples. In addition, after each determination of a spiked sample, an analysis of acetonitrile was performed to eliminate the possible influence of PAHs added to the meat on the result of the sample analyzed in sequence. Each batch ended with measurements of PAH standard solutions with concentrations of 1 ng, 0.5 ng and 0.05 ng in 10 µL of acetonitrile. The results of these determinations were used to determine the inter-day precision.

The intra-day precision (repeatability) of the HPLC-FLD method, expressed as relative standard deviations (RSD) of five results obtained on one day for samples with concentrations of 1 ng, 0.5 ng and 0.05 ng of PAHs (in 10 µL of acetonitrile), ranged from 0.21% (for BaA) to 2.63% (for BbF). The reproducibility (inter-day precision), conducted over five days, was in the range of 0.23% (for BaA) and 3.37% (for Chr).

The purification procedure used, which included a hydrolysis step, solid-phase extraction and silica gel adsorption column chromatography, made it possible to isolate PAH fractions free of fat and other components present in the matrix of the meat samples [49]. Analyses of non-spiked meat samples and spiked with standards were carried out to avoid the influence of the matrix on the position of the peaks on the chromatogram, and to estimate the probable losses of PAHs during the multi-step analytical procedure of PAH isolation. Recoveries were calculated with the use of the following equation:

$$\mathrm{Recovery}=\frac{C_{1}-C_{2}}{C}\cdot 100\%$$

where *C*_1_ is the PAH concentration in a meat sample spiked with standard (ng/g), *C*_2_ is the PAH concentration in a non-spiked meat sample (ng/g) and *C* is the amount of standard added to a spiked sample (ng/g). The recoveries, presented in Table 3, ranged from 61.2% (BaP, meat sample spiked with 10 ng/g) to 96.1% (Chr, meat spiked with 40 ng/g). The recovery rates are comparable to the values found by authors of other studies [49,52] when determining PAHs in meat samples. These results meet the performance criteria for methods for analyzing concentrations of polycyclic aromatic hydrocarbons in food products. These criteria are used to determine the concentration of the four polycyclic aromatic hydrocarbons: benzo(a)pyrene, benzo(a)anthracene, benzo(b)fluoranthene and chrysene. According to the recommendations of the European Commission, the recovery of these compounds should be in the range of 50–120% [28].

### 2.7. Identification of PAHs by Gas Chromatography—Mass Spectrometry/Mass Spectrometry (GC-MS/MS)

The extracts remaining after quantitative determination by HPLC-FLD technique were used for GC-MS/MS analysis to confirm the presence of PAHs in meat samples. A gas chromatograph, Trace 1310 Thermo Scientific (Milano, Italy) with a triple quadrupole mass spectrometer, TSQ 9000 (Thermo Scientific, San Jose, CA, USA), was applied. The compounds were separated on a TG-5MS GC capillary column (30 m × 0.25 mm; film thickness: 0.25 µm) from Thermo Scientific (Waltham, MA, USA). Conditions for the analysis of PAHs were as follows: split-less injection (2 min), helium flow rate of 1 mL/min; GC temperature program: 70 °C (2 min), 5 °C/min to 320 °C (5 min), temperature of injector 270 °C, interface 320 °C, ion source 320 °C; electron ionization (EI) 70 eV. Extract solutions were injected into the column at 1 µL using a TriPlus RSH auto sampler (Thermo Fisher Scientific, San Jose, CA, USA). The analyses were performed in the full scan mode (mass range 50–550 Da) and using the selective reaction monitoring (SRM) mode. The precursor ions and product ions were selected as a result of an automatically performed experiment for a PAH standard solution. The mass spectra of PAHs are dominated by their molecular ion [M]+. The identification of PAHs involved comparing mass spectra and retention times of compounds identified in extracts from food samples and standards. Identification was performed using the mass spectral databases Mainlib and NIST (National Institute of Standards and Technology in USA) and Chromeleon software (version 7.2.10).

### 2.8. Statistical Analysis

Basic descriptive statistical parameters, such as mean, standard deviation and relative standard deviation, were used to show the results of PAH determination. Statistical calculations were performed using the Statistica 13.3 platform for statistical calculations (TIBCO Software Inc., Palo Alto, CA, USA). First, normality tests were performed: Kolmogorov–Smirnov, Kolmogorov–Lilliefors and Shapiro–Wilk for all variables. Then, tests for two means were performed (ANOVA for two means). Non-parametric Kruskal–Wallis and median tests were also performed. Then, tests were carried out to verify the null hypothesis about the identity of the probability distributions of the content of specific chemical compounds in individual types of meat. The significance level of the test was assumed to be 0.05, and the test itself was carried out using the following tests: U Mann–Whitney, Wald–Wolfowitz series and Kolmogorov–Smirnov.

### 2.9. Determination of Dry Mass and Water Containment in Fruits

Dry matter content was determined at 105 °C. One-gram portions of crushed fruit placed on Petri plates were dried to a constant weight. Drying was carried out using the laboratory oven Venticell (BMT Medical Technology s.r.o., Brno, Czech Republic). Each sample was performed five times and the results were averaged [53].

## 3. Results and Discussion

### 3.1. PAHs in Meat Samples

Data from the HPLC-FLD analysis of PAHs in the examined meat samples coming from dishes prepared without and with dried fruits are presented in Table 4. 

The PAH concentrations were from “below the LOQ” (BaP in meat with cranberries and DiBahA in meat with prunes) to 2.01 ng/g (Chr in meat without additives). The aim of this research was to determine the content of PAHs, for which the maximum levels of concentration in selected foodstuffs were specified in Regulation No 835/2011 of the European Commission [28]. These include compounds from the PAH4 group, i.e., BaA, Chr, BbF and BaA. In addition, three other compounds from the PAH8 group, namely BkF, DiBahA and BghiP, were included in this study. Unfortunately, due to the lack of an appropriate standard, indeno(1,2,3-cd)pyrene was not determined. PAH4 are compounds that have been recognized by the EFSA Panel on Contaminants in the Food Chain [27] as optimal indicators of exposure to PAHs in food. EFSA also stated that a system of PAH8 would not bring much added value compared to a system of PAH4 [27].

The thermally meat cooking by using roasting bags is an increasingly popular and simple cooking technique. Moreover, in Poland, the tradition of preparing meat dishes stuffed and baked with fresh or dried fruits is very common. Recipes combining the old Polish tradition of using fruit as a meat filling and the use of a modern food preparation technique, in this case baking in an electric oven with the use of roasting bags, are proposed in various popular cooking programs and cookbooks. We found no information in the scientific literature on the effects of dried apricots, cranberries and prunes on the formation of PAHs. 

Many factors may cause the formation of PAHs, so our examination was planned in such a way that, apart from the fruit used as the pork filling, the repeatability of all other parameters of dishes preparation was maintained. In order to examine only the effect of dried cranberries, prunes and apricots on the formation of PAHs in meat, the dishes were made from pork joints that came from the same animal. The cooking conditions were the same. The results of various studies show that the type of meat [54,55,56,57,58], fat content in the meat [52,59,60], water [59], oils used for frying [61,62], spices added [38,44,63] and cooking methods [54,57,58] can influence the concentration of PAHs.

Oil may be an additional source of PAHs [18,62]. Frying oil interference was eliminated by roasting meat in a roasting bag without oil. Moreover, the conditions of heat treatment of roasting meat in a roasting bag are more similar to the conditions of stewing meat. Despite the long bake in the oven, the meat in the roasting bag did not brown very much.

The summary content of the seven determined PAHs in meat without fruits was 7.38 ng/g and was 6.14 ng/g in terms of PAH4 (BaA, Chr, BbF, BaP). Thus, PAH4 accounts for 83.2% of the total content of the seven assayed compounds, which supports the thesis that PAH4 constitutes the majority of compounds compared to PAH8 [27]. The concentration of carcinogenic BaP in these meat was 0.87 ng/g.

The methods of thermal processing by roasting meat using an electric oven or stewing in sauce, as well as cooking meat, allows one to obtain dishes with lower concentrations of PAHs than in dishes prepared by barbecuing or grilling, mainly charcoal grilling [32,38,40,48,49,52,64,65,66]. The PAHs results obtained in this study are of the same order as in dishes prepared by pan frying, stewing in sauce or boiling meat [47,48,55,65,66].

Duedahl-Olesen et al. studied 203 commercially barbecued meat samples and reported a very high level of BaP and PAH4, up to 63 ng/kg and 195 ng/kg, respectively, in a pork tenderloin [56]. Cordeiro et al. detected BaP and PAH4 in charcoal-grilled pork loin at levels of 3.4 and 31.5 ng/g of meat, respectively [32]. Onopiuk et al. [38] measured the concentration of 12 PAHs in grilled pork. The meat was pre-soaked for 24 h in water with 5% NaCl. The BaP and PAH4 contents in these samples were 1.7 and 24.8 ng/g, respectively. The grilled meat contained high amounts of DiBahA (38.1 ng/g) and Chr (18.8 ng/g). In the work of Chen and Lin, the concentration of BaP in samples of grilled duck was determined to be in the range from 0 to 9.5 ng/g. However, this compound was not present in duck meat roasted in an electric oven [64]. Olatunji et al. studied the concentrations of BkF, BaP, IP and BghiP in grilled and boiled meat. PAH concentrations in grilled meat ranged from 1.4 ng/g (BkF) to 4.0 ng/g (IP), while those in boiled meat were about 1 ng/g (BaP, BkF BghiP) [65]. Coroian et al. [66] evaluated PAH levels in pork shoulders, ham and bacon after various thermal processes (cooking, roasting and curing). The highest levels were found for phenanthrene (up to 35.7 ng/g) in meat prepared by roasting and curing, while the lowest levels were determined for BaP (0.35 ng/g, boiled ham) and BkF (0.27 ng/g, boiled pork meat). The author of this paper, Janoszka, determined BaP in grilled pork neck to be 2.3 ng/g [48]. In another study, chops and collars prepared from pan-fried pork loin contained less BaP; 1.6 and 1.1 ng/g, respectively. The total content of six PAHs (BaA, BbF, BkF, DiBahA and BghiP) was 7.8 and 6.4 ng/g in chop and collar samples, respectively, and therefore was similar to the results of this study [47].

In contrast, Samiee et al. [58] when investigating 16 PAHs, including PAH4, in 40 samples of processed meat (sausages and burgers) collected from an Iranian market, found higher levels of total PAHs and PAH4 in fried meat (PAH = 2.35 ng/g) than in grilled meat (PAH = 1.47 ng/g). Of the PAH4 group, the hydrocarbon with the highest concentration (as in our work) was Chr, containing 1.61, 1.16 and 1.14 ng/g in fried, grilled and uncooked meat, respectively. It is noteworthy that the highest number of PAHs were determined in products with high (90%) meat content. BaP (0.21 ng/g) was determined only in fried products with the highest percentage of meat. BaP was also not detected in the roasted pork jerky samples studied by Lai et al. [45], but ten other PAHs were determined in these samples, with a total of 72.6 ng/g, including only three PAH8 compounds: BaA (1.8 ng/g), BghiP (3.6 ng/g) and Chr (4.9 ng/g). The compound found in the highest concentration was pyrene (38.9 ng/g). 

The cited results indicate that thermal treatment of meat by grilling or frying is usually associated with obtaining a dish with relatively high PAH content. The individual composition of the compounds can vary depending on the type of meat and heat treatment, but the contents remain in the concentration range of the order of ng/g.

One of the main factors contributing to PAH formation in grilled meat is the fumes from the incomplete combustion of the fat which drips onto the fire [34,67]. This is not the case when pan frying or using a roasting bag. However, there are opinions that bisphenol A (BPA), a chemical that can cause cancer, may be released during the use of a roasting bag [68]. Research by Savas et al. showed that the levels of BPA in meat samples are not high enough to pose a risk to human health [69].

### 3.2. Influence of Dried Fruits on PAHs

**Figure 2:**
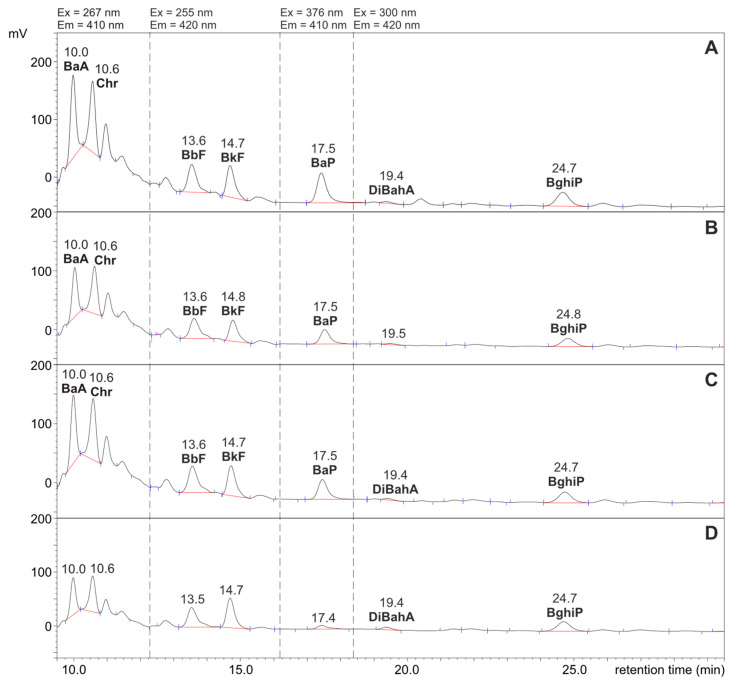
HPLC-FLD chromatograms of fractions containing PAHs separated from roasted pork loin samples. (**A**): Control sample (pork meat prepared without additives); (**B**): pork meat with prunes; (**C**): with apricots; (**D**): with cranberries. In each case, 10 µL of 500 µL was injected.

The use of dried fruits (apricots, cranberries and prunes) in the amount of 200 g/kg as an addition to thermally processed meat led to a reduction in the content of PAHs in these dishes. The HPLC-FLD chromatograms shown in Figure 2A–D were obtained during the determination of PAHs in fractions isolated from meat samples with and without fruit. In order to illustrate the changes in PAH concentrations caused by the added dried fruit, the same scale of the ordinate axis (mV intensity) was used in all chromatograms. 

The concentration of PAHs in the samples of pork with fruits were in the range from not quantifiable (DiBahA in meat with prunes and BaP in meat with cranberries) to 1.2 ng/g (Chr and BbF in pork loin with apricots). Recent research on the presence of PAHs in dried fruits available on the Polish market (including prunes, apricots and cranberries) have shown that they do not contain compounds from PAH4 and PAH8 [70]. This is consistent with our results, because none of the meat dishes prepared by stuffing 1 kg of pork with 200 g of fruit showed an increase in the concentration of PAHs. 

Table 4 presents the results for the individual PAHs determined, the total contents of seven PAHs and of PAH4 (i.e., BaA, Chr, BbF and BaP). The table also shows the calculated percentages of reduction in the concentration of PAHs in dishes prepared with dried fruit compared to the dish without these additives (control sample). The strongest inhibitory effect on the formation of PAHs in meat was noted in the dish with the addition of cranberries. Pork loin roasted and stuffed with this fruit contained 58% less of all seven PAHs and 60% less if only compounds from the PAH4 group were included. It is worth noting that under the influence of cranberries, the concentration of BaP decreased to a value lower than LOQ; therefore, this compound was not determined in this meat sample. Under the influence of prunes and apricots, the concentration of the seven total determined PAHs decreased by 48% and 34%, respectively. These changes for PAH4 were similar and amounted to 47% (for prunes) and 37% (for apricots), respectively. The reduction in concentrations of individual PAHs in samples from meat roasted with fruit compared to the control sample was statistically significant in most cases (*p* < 0.05). The exception was the meat sample with apricots, in which the BghiP concentration was not statistically different from that of the control. 

A review of the literature shows that meat dishes prepared with natural additives rich in antioxidants often contained less muta- and carcinogenic compounds from the PAHs group [43]. The studies mainly focused on grilled dishes prepared with marinades containing various ingredients, including beer [39,40], tea extracts [41,71] or solutions of compounds found in tea [72], different vinegars, applied as a seasoning [32] and spices and herbs [36,38,44,73]. So far, few studies have been published on the impact of fruits or ingredients obtained from them on the formation of PAHs [32,46].

In some studies, apart from PAHs, antioxidant activity and the composition of natural additives were also determined. The aim of these studies was to determine the influence of additives on the mechanism of PAH formation during the grilling of meats [39,40,42,72]. 

The inhibitory effect of marinades on PAHs in charcoal-grilled meat has been shown to increase as their radical scavenging activity increases [39]. The main reasons for the reduction in PAHs in meat were the antioxidant capacity and the content of polyphenols in natural food extracts (e.g., green tea, grape seeds and rosemary) [71].

Studies on the impact of beer marinades and the phenolic compounds found in them on the formation of PAH8 confirmed that free radicals and phenolic compounds affect the mechanisms of PAH formation and inhibition, respectively. Among the antioxidants determined in beer, two phenolic compounds were the most effective in reducing the PAH8 concentration: homovanillic acid (PAH8 reduction by 58%) and ferulic acid (57%). The authors noted that the inhibitory effect of phenolic acids is greater than that of other phenolic compounds. This is due to the presence of a carboxyl group that exhibits different electron transfer mechanisms or rates than phenolic hydroxyl groups [40]. 

A study on the effect of marinades containing selected phenolic acids on the formation of PAHs in charcoal-grilled chicken wings showed that a concentration of protocatechuic acid of 3 mg/L had the greatest inhibitory effect (40.3%) on PAH8, followed by gallic and ferulic acids (26%). This effect on the concentration of BaP was different for each of the acids and was the strongest (36%) for ferulic acid [42]. However, these studies also showed that the inhibition of PAH formation was not significantly correlated with the antiradical activity of the marinades.

In a study of the effect of a marinade containing fruit-derived ingredients, i.e., apple polyphenols, on the formation of PAHs in pork grilled on charcoal, their inhibitory effect on the formation of PAHs was shown to depend on their concentration [46]. Among the antioxidants determined in the solution of apple polyphenols, procyanidins (45%) and chlorogenic acid (31%) dominated. Under the influence of the addition of 0.20% apple polyphenol, the inhibition of PAHs and BaP was 53% and 100%, respectively, compared to the control samples. It was also noted that the addition of apple polyphenols inhibited the oxidation of fatty acids. Inhibition of lipid oxidation could reduce the cyclization reaction of PAH precursors, and thus could reduce the concentration of PAHs in grilled pork [46]. 

The dried fruits that were used as fillings in the pork loin dishes of our study may contain various biologically active compounds, including antioxidants [74,75,76]. The research on ingredients marked in prunes, apricots and cranberries available on the Polish market shows that phenolic acids are the main part of the total composition of polyphenolic compounds found in dried fruits [75]. The content of these acids determined in prunes is about 300 mg/100 g of the product, about 160 mg/100 g in cranberries and about 130 mg/100 g in apricots.

The previously cited literature data on the influence of different marinades ingredients on the concentration of PAHs in heat-treated meats indicate that phenolic acids are often mentioned among the factors inhibiting PAH formation [40,42,46]. 

Dried fruits contain gallic, chlorogenic, protocatechuic and ferulic acids. All these are compounds with a proven ability to reduce PAHs [75,76,77,78]. The concentrations of these phenolic acids in dried fruit are in the order of mg/100 g of the product. According to the results of fruit determinations from Polish stores, the dominant acids in prunes are gallic acid (about 95 mg/100 g) and chlorogenic acid (about 117 mg/100 g). Apricots contain less of these acids (gallic acid, about 60 mg/100 g, and chlorogenic acid, about 22 mg/100 g). The highest amount of chlorogenic acid was found in cranberries (about 135 mg/100 g) [75]. The content of gallic acid in dried cranberries, according to Dorofejeva et al., is about 1.8 mg/100 g dry matter [77]. Vanillic acid was found in prunes at an amount of 3–10 µg/100 g [78]. Prunes also contain small amounts of ferulic acid (about 1 mg/100 g) [75]. 

Comparing the results of the determination of individual PAHs in meat samples with added fruit (Table 4), it can be seen that the concentrations of Chr, BbF, BkF and BghiP for meats with cranberries and prunes are similar to each other and are not statistically different (*p* > 0.05). The percentage reduction in the concentrations of these compounds relative to the control sample is also similar. A common feature in both of these dried fruits is the high content (above 100 mg/100 g versus about 20 mg in apricots) of chlorogenic acid [75].

In addition to phenolic acid, dried fruit also contains small amounts of antioxidant vitamins, including vitamin C and E (α-tocopherol) [74,76]. According to the data from the literature, the content of vitamin C in prunes and apricots may be about 1 mg/100 g, while the content of α-tocopherol in apricots may be slightly higher (about 4 mg/100 g) [74,76]. The content of vitamin C in cranberries, depending on the method of drying the fruit, can range from a few mg to about 20 mg/100 g [79]. Studies conducted in a heated meat model system showed that 200 µg/kg α-tocopherol led to a decrease in BaA, Chr, BbF, IP, BghiP and DiBahA, but an increase in BaP (from 1.17 to 4.95 ng/g) [59]. On the other hand, marinating meat with a solution containing 0.02% of ascorbic acid reduced the content of BaA, BaF and BaP (100%) in barbecued pork [46]; this is similar to the results of our research on pork with cranberries, the addition of which to meat resulted in a 58% reduction in PAH4 and 100% reduction of BaP.

Dried fruits can also be a valuable source of another antioxidant, which is quercetin. Apricots contain about 25 mg/100 g of this compound, while prunes and cranberries contain small amounts of this antioxidant (less than 0.5 mg/100 g) [75]. The use of a marinade with quercetin contents of 100 and 500 mg/kg lowered the concentration of PAH4 in grilled sirloin pork by almost three times. Reductions in BaP (by 20%), Chr and BbF (60%), and BaA and BghiP (100%) were also observed [80].

The results of our study indicate that stuffing meat with cranberries had the strongest inhibitory effect on the formation of PAHs (Table 4). A review of the literature shows that these fruits contain more vitamin C and chlorogenic acid than prunes and apricots. Both ingredients have also been shown to be effective in lowering PAHs in studies on the effect of apple polyphenol (containing 31% chlorogenic acid) and other antioxidants, including ascorbic acid, on the formation of PAHs in barbecued pork [46]. 

The amount of antioxidants introduced into the dish by using 200 g of dried fruit filling in our experiment to 1 kg of pork loin could be high. The dried fruits used as a filling for the meat were placed in a hole cut in the loin in constant contact with the meat before and through thermal treatment in the electric oven. During roasting, antioxidants may have been released from the fruit along with steam, which, by entering the meat, could inhibit the formation of PAHs.

The results of estimating the antioxidant properties of dried fruits used as fillings for pork loin by determining the iron reduction parameter (FRAP) and the reduction in the 2,2-diphenyl-1-picrylhydrazyl radical (DPPH) by antioxidants were previously reported in our study [81]. The results showed that the activity of cranberry was about three times lower than for prunes and two times lower than for apricot; however, it was observed that the addition of cranberry had a stronger effect on reducing PAH4 concentrations than prunes and apricots. Similar results were recorded when using different vinegars sprayed on meat before charcoal grilling [32]. Elderberry vinegar was the most effective in inhibiting PAH4 formation with an inhibition of 82% (relative to the control). However, the authors observed that using white wine vinegar, whose antioxidant capacity and total phenolic content were many times lower than in the case of elderberry vinegar, had only a slightly weaker inhibitory effect on PAHs (by 3%) [32]. Additionally, research on the effects of phenolic compounds occurring in green tea showed that although these compounds inhibit PAH formation in charcoal-grilled chicken wings, there is no relationship between the reduction in the content of phenolic compounds in the investigated samples and the formation of PAH8 [72]. 

The mechanism of PAH formation in thermally processed foods is complicated and not fully understood. The literature states that in the first stage, pyrolysis occurs, during which molecules of organic food ingredients are easily fragmented under the influence of high temperature. In the second stage, pyrosynthesis takes place, during which the free radicals formed in the first stage can form PAHs [29,82]. The probable mechanism of action of food additives rich in antioxidants consists of the deactivation of free radicals, which are intermediate products of the compounds formed.

Bao et al. studied the formation of free radicals in roasted meat using electron spin resonance [82]. Studies have shown a reduction in the formation of radicals when roasting poultry, beef and pork after adding 0.03% polyphenols in tea. In the same study, when 0.03% rosemary extract was added, no significant reduction was observed. The authors also paid attention to the amount of water in roasted meat. Water has been shown to delay the formation of radicals in roasted meat. As stated in the methodology, the weight of roasted meat obtained from 1 kg of pork loin prepared with the addition of fruit was several % higher than the weight of the control sample (without additives). The determined water content in the fruit used as the filling was 24.3% (cranberry), 25.8% (apricot) and 36.5% (prune). These values are close to the literature data [74]. It is probable that the water contained in the fruit filling the meat, remaining in constant contact with the meat during roasting, accumulated in the meat. According to the results of the work of Bao et al., a greater amount of water in the dish could have had an impact on slowing radical generation, and thus on the formation of PAHs as a result of radical processes. Studies by other authors also confirm that water is an important factor affecting the inhibition of PAH formation in meat model systems [59].

The results of many studies indicate a significant inhibitory effect of natural additives on the formation of polycyclic aromatic hydrocarbons in thermally treated meat. However, the mechanisms of PAH inhibition by antioxidants is complex, and it is probable that the radical scavenging activity of polyphenols was not the only influence on the process. Additionally, phenolic profiles, as well as reactions that can occur between meat ingredients and components of natural additives, can affect the formation of PAHs [32]. Synergistic or antagonistic effects of various antioxidant compounds were also observed by studying their effects on the synthesis of other carcinogens formed by radical processes in meat, i.e., heterocyclic aromatic amines [83].

### 3.3. Identification of PAHs in Meat Samples Using GC-MS/MS Technique

After the quantification of PAHs using the HPLC-FLD technique, the remaining extract solutions were used for qualitative determinations using the GC-MS/MS technique. These analyses were performed to confirm the presence of PAHs in meat samples and consisted of comparing retention times and mass spectra recorded for PAH standards and extracts isolated from meat samples. In the Supplementary Materials, Figure S2 presents the exemplary mass spectrum of BaP and chrysene recorded for the extract isolated from the meat roasted without additives (control sample). Spectra from the Mainlib and NIST databases are also shown for comparison. 

**Figure 3:**
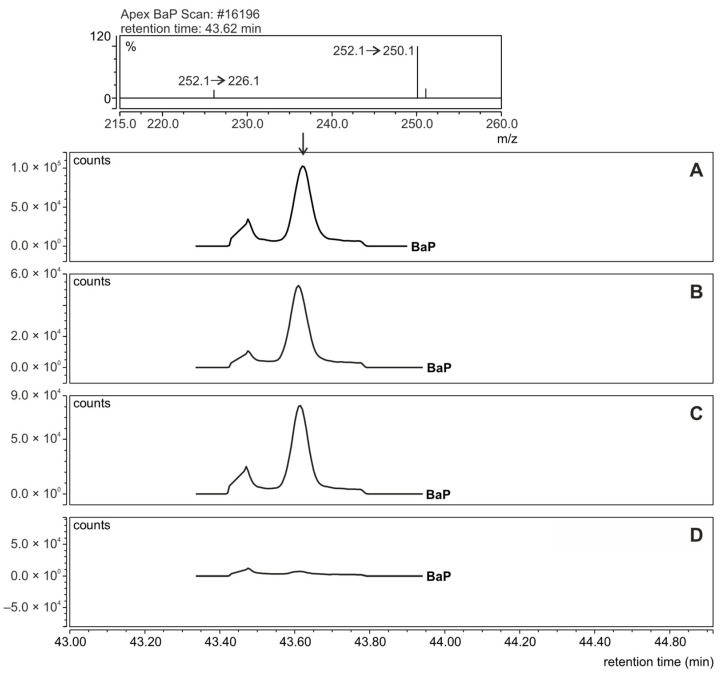
GC-MS/MS chromatograms recorded in selective reaction monitoring (SRM) mode for the detection of BaP in fractions isolated from meat samples. (**A**): Control sample (pork meat prepared without additives); (**B**): pork meat with prunes; (**C**): with apricots; (**D**): with cranberries. The chromatograms corresponds to the fragmentation reaction of the precursor ion (*m*/*z* = 252.1) to the product ion (*m*/*z* 250.1). The collision energy for this reaction was 35 eV.

GC-MS/MS chromatograms recorded in selective reaction monitoring (SRM) mode during BaP detection in fractions isolated from meat samples are shown as an example in Figure 3. By performing this analysis, it was possible to confirm the absence of BaP in meat prepared with cranberries. GC-MS/MS analysis confirmed the presence of the other six PAHs quantified by HPLC-FLD in meat roasted without and with added fruits. 

### 3.4. Reduction in Exposure to PAHs through Consumption of Meat Dishes Prepared with Natural Additive

The concentrations of polycyclic aromatic compounds in pork meat prepared by using a roasting bag were not high. The total content of all seven determined PAHs was only 7.4 ng/g, and BaP around 0.9 ng/g. These concentrations are much lower than the maximum levels set by the European Commission’s Regulation No. 835/2011 for “heat treated meat and heat treated meat products sold to the final consumer”, which for PAH4 and BaP are 30 and 5 ng/g, respectively [28].

Meat and meat products are the basic source of amino acids for humans; therefore, they cannot avoid exposure to the harmful compounds formed during thermal processing. According to the WHO report from 2015, in many countries around the world, a person eats over 150 g of meat per day [7]. It follows that the exposure to carcinogenic compounds through the diet may be even several dozen to several hundred ng/day of PAHs alone with the consumption of a 100 g portion of meat. For this reason, any processes or recipes that allow the formation of carcinogenic compounds in high-protein products to be inhibited, and that can also be used in households, are valuable. Our research shows that the use of dried fruit as a filling for pork roasts allowed us to obtain dishes with a lower content of the determined seven PAHs in the range of 34% to 58%.

In addition, the consumption of natural plant supplements has a positive effect on human health. This applies to spices, natural marinades and the fruit additives that were used in this work.

Fruits contain numerous ingredients that have a positive effect on the human body. These are vitamins, minerals, polyphenolic compounds and fiber, which can protect against gastrointestinal cancers. In addition, the products of plant metabolism have antibacterial and anti-inflammatory properties [74,75].

## 4. Conclusions

The thermal processing of high-protein foods can lead to the formation of polycyclic aromatic hydrocarbons. Seven PAHs (BaA, Chr, BbF and BaP, BkF, DiBahA and BghiP) were determined in pork loin roasted in an electric oven using a roasting bag. The total PAH content was 7.4 ng/g. 

It was possible to reduce this concentration by roasting the pork loin with the addition of dried fruits used as stuffing for the meat. The total PAH content decreased by 35%, 48% and 58% when the meat was prepared with apricots, prunes and cranberries, respectively. Cranberries had the strongest inhibitory effect on the formation of PAHs, reducing BaP content by 100%. 

None of the meat dishes tested had concentrations of PAH4 and BaP above the maximum levels of 30 and 5 ng/g, respectively, set by the European Commission’s regulation for heat-treated meat (No. 835/2011).

The use of natural additives, in the form of dried fruits, in thermally processed meat results in dishes with a lower content of compounds harmful to health. The results obtained can be an indication of how to minimize human exposure to dietary carcinogenic compounds through simple methods of preparing meat dishes.

It should be added, however, that in this study only one type of meat was used to assess the effect of the addition of fruit alone on the formation of PAHs. With awareness that the process of PAH formation depends on many parameters, including fat content and cooking methods, it seems justified to extend the research to various meat samples coming from different animals in the future. In addition, it would be worth investigating the relationship between the amount and composition of various fruits (dried, fresh) added to meats and their potential to reduce PAHs.

## Figures and Tables

**Table 1:** Names and structures of the PAHs determined in this study.

Name	Structure	Name	Structure
Benzo(a)pyrene BaP	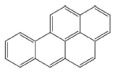	Dibenzo(ah)anthracene DB(ah)A	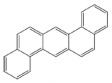
Chrysene Chr	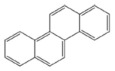	Benzo(k)fluoranthene BkF	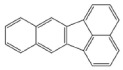
Benzo(b)fluoranthene BbF	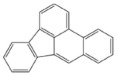	Benzo(ghi)perylene BghiP	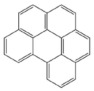
Benzo(a)anthracene BaA	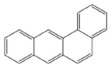		

**Table 2:** Data from PAHs determination by HPLC-FLD method.

PAH	Abbreviation	Calibration Curve Concentration Range (ng/mL)	Regression Coefficients *r*	LOQ ^1^ (ng/mL)	LOD ^2^ (ng/g)	LOQ ^1^ (ng/g)
Benzo(a)anthracene	BaA	0.5–200	0.9999	0.30	0.003	0.010
Chrysene	Chr	0.5–200	1.0	0.45	0.004	0.012
Benzo(a)pyrene	BaP	0.5–200	1.0	0.45	0.004	0.012
Benzo(k)fluoranthene	BkF	0.5–200	1.0	0.30	0.003	0.010
Dibenzo(ah)anthracene	DiBahA	0.5–200	1.0	0.45	0.004	0.012
Benzo(ghi)perylene	BghiP	0.5–200	1.0	0.45	0.004	0.012
Benzo(b)fluoranthene	BbF	1.0–200	1.0	0.6	0.006	0.020

^1^ LOQ—limit of quantification. ^2^ LOD—limit of detection.

**Table 3:** Recoveries of PAHs spiked in meat samples (*n* = 9).

PAH	Recovery (%) and RSD ^1^ (%) for Spiking Level:	
	10 ng/g	40 ng/g
BaA	87.2 (14.4)	89.4 (14.9)
Chr	88.6 (15.9)	96.1 (8.9)
BbF	83.9 (10.5)	86.8 (11.7)
BaP	61.2 (13.6)	67.9 (12.7)
BkF	73.5 (9.9)	79,2 (11.3)
DiBahA	72.1 (12.8)	73.8 (7.8)
BghiP	75.9 (8.1)	75.3 (8.5)

^1^ RSD—relative standard deviation.

**Table 4:** PAH concentration (ng/g of meat) and the inhibitory effect (%) of dried fruits on PAH content in pork meat roasted with and without additives. The results are presented as means ± standard deviations (SD). They correspond to two HPLC analyses of the fractions obtained from three repeated extractions of each meat sample (n = 6). Different letters (a, b, c, d) in each row indicate statistically significant differences (*p* < 0.05).

PAH	Concentration ^1^ (ng/g) and Inhibition (%) in Pork Loin Samples			
	Without Additives (Control)	With Prunes	With Apricots	With Cranberries
BaA	1.42 ± 0.10 ^a^	0.83 ± 0.05 ^b^ (41.5%)	0.92 ± 0.06 ^c^ (35.2%)	0.57 ± 0.04 ^d^ (59.9%)
Chr	2.01 ± 0.17 ^a^	1.14 ± 0.06 ^b,c^ (43.3%)	1.22 ± 0.12 ^b^ (39.3)	1.03 ± 0.06 ^c^ (48.8%)
BbF	1.84 ± 0.22 ^a^	0.91 ± 0.13 ^b^ (50.5%)	1.24 ± 0.12 ^c^ (32.6%)	0.84 ± 0.08 ^b^ (54.4%)
BaP	0.87 ± 0.07 ^a^	0.35 ± 0.03 ^b^ (59.8%)	0.48 ± 0.07 ^c^ (44.8%)	n.q. ^2^ (100%)
BkF	0.43 ± 0.06 ^a^	0.25 ± 0.04 ^b^ (41.9%)	0.36 ± 0.04 ^c^ (16.3%)	0.26 ± 0.01 ^b^ (39.5%)
DiBahA	0.27 ± 0.04 ^a^	n.q. ^2^ (100%)	0.12 ± 0.02 ^b^ (55.6%)	0.06 ± 0.004 ^c^ (77.8%)
BghiP	0.54 ± 0.04 ^a^	0.34 ± 0.05 ^b^ (37.0%)	0.50 ± 0.09 ^a^ (7.4%)	0.35 ± 0.03 ^b^ (35.2%)
PAH4 ^3^	6.14	3.23 (47.4%)	3.86 (37.1%)	2.44 (60.3%)
Total PAHs ^4^	7.38	3.82 (48.2%)	4.84 (34.4%)	3.11 (57.9%)

^1^ Concentrations have not been corrected for recovery values. ^2^ n.q.—not quantified (below the LOQ). ^3^ PAH4—BaA, Chr, BbF, BaP. ^4^ Total PAHs—BaA, Chr, BaP, BbF, BkF, DiBahA, BghiP.

## Data Availability

The data presented in this study are included in this article.

## Supplementary Materials

The following supporting information can be downloaded at: https://www.mdpi.com/article/10.3390/ijerph20064922/s1, Figure S1: Scheme of analytical procedure for separation and determination of polycyclic aromatic hydrocarbons (PAHs) in meat samples; Figure S2: GC-MS mass spectra of BaP (A) and chrysene (C) recorded for the extract isolated from the pork meat roasted without additives (control sample). Spectra from the Mainlib and NIST databases are also shown for comparison: BaP (B) and chrysene (D).
