# Supplementary material for: Polycyclic Aromatic Hydrocarbons (PAHs) in Roasted Pork Meat and the Effect of Dried Fruits on PAH Content
Source: Int J Environ Res Public Health. 2023 Mar 10;20(6):4922. doi: 10.3390/ijerph20064922 (PMC10049194; doi:10.3390/ijerph20064922)
Supplement: Supplementary file 1 [file ijerph-20-04922-s001.zip › Figure S1 Bulanda.pdf]

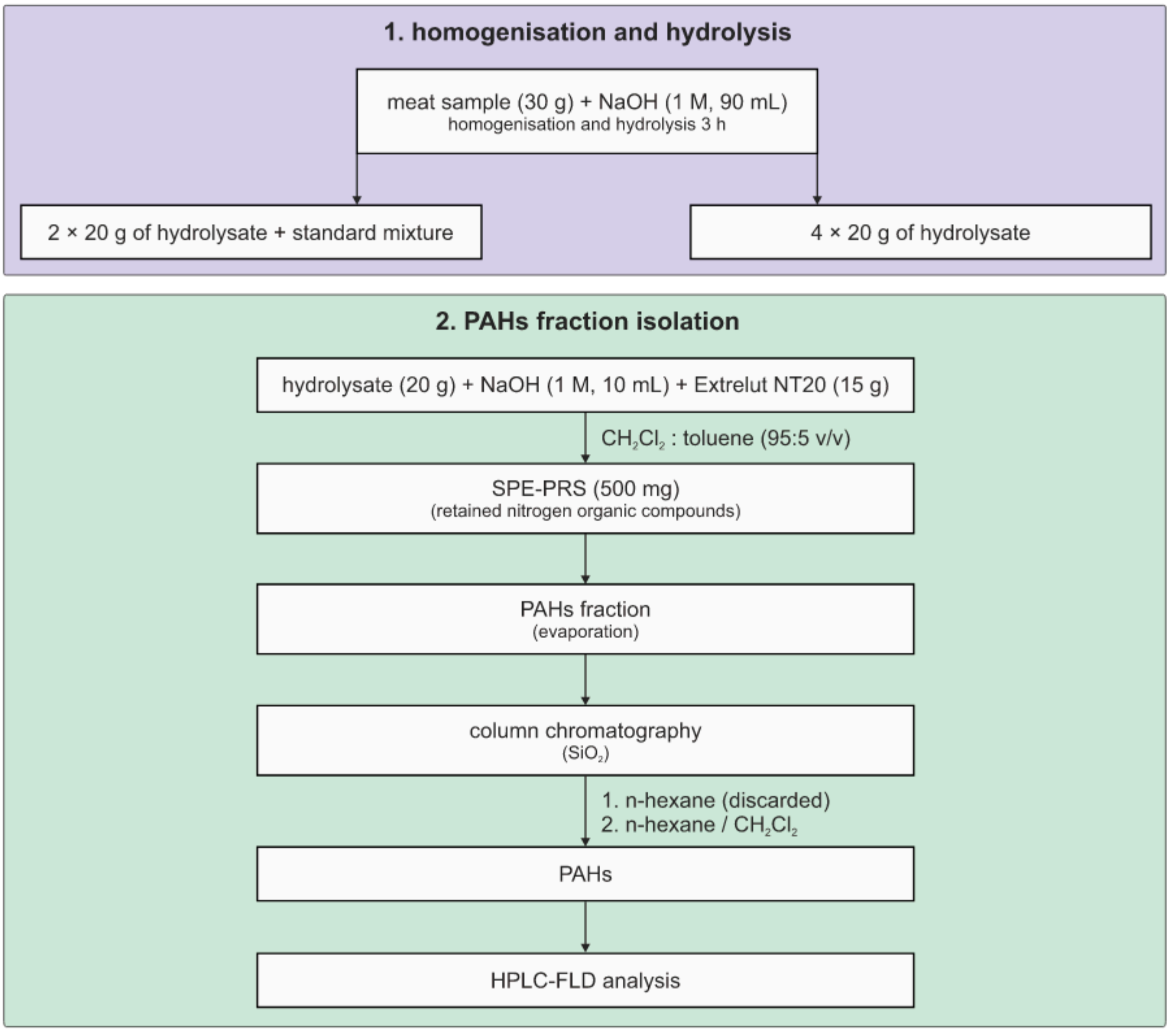

Figure S1. Scheme of analytical procedure for separation and determination of polycyclic aromatic hydrocarbons (PAHs) in meat samples.

Abbreviations:
SPE - Solid phase extraction
SPE-PRS - SPE columns filled with propyl sulfonic acid (PRS)
HPLC-FLD – high performance liquid chromatography with fluorescence detector
Standard mixture contained PAHs standards
