# Supplementary material for: Polycyclic Aromatic Hydrocarbons (PAHs) in Roasted Pork Meat and the Effect of Dried Fruits on PAH Content
Source: Int J Environ Res Public Health. 2023 Mar 10;20(6):4922. doi: 10.3390/ijerph20064922 (PMC10049194; doi:10.3390/ijerph20064922)
Supplement: Supplementary file 1 [file ijerph-20-04922-s001.zip › Figure S2 Bulanda.pdf]

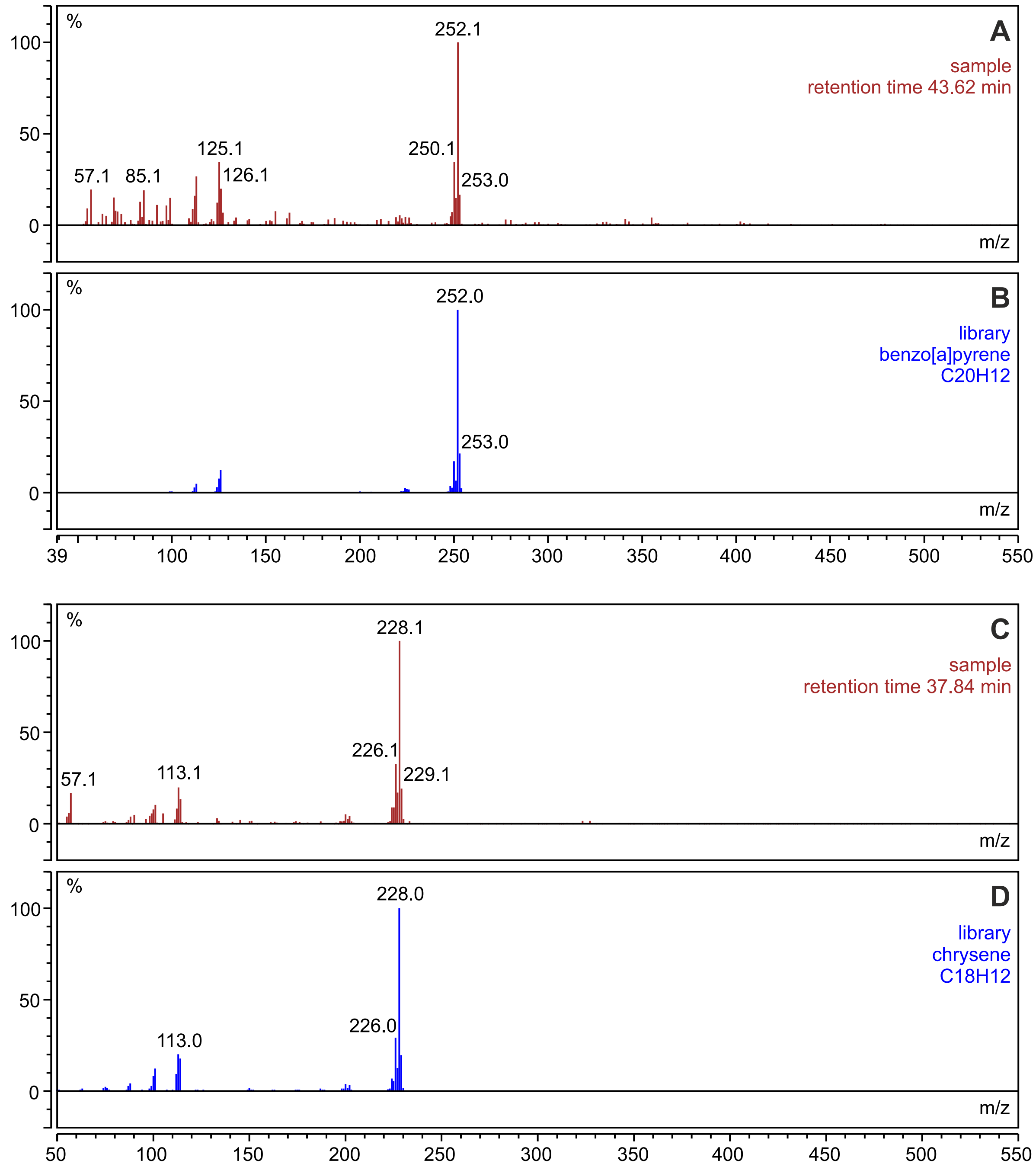

Figure S2. GC-MS mass spectra of BaP (A) and chrysene (C) recorded for the extract isolated from the pork meat roasted without additives (control sample). Spectra from the Mainlib and NIST databases are also shown for comparison: BaP (B) and chrysene (D).
